# 1-(4-Bromo­phen­yl)-2-ethyl­sulfinyl-2-(phenyl­selan­yl)ethanone monohydrate

**DOI:** 10.1107/S1600536811012712

**Published:** 2011-04-13

**Authors:** Julio Zukerman-Schpector, Carlos A. De Simone, Paulo R. Olivato, Carlos R. Cerqueira, Edward R. T. Tiekink

**Affiliations:** a Department of Chemistry, Universidade Federal de São Carlos, 13565-905 São Carlos, SP, Brazil; b Instituto de Química e Biotecnologia, Universidade Federal de Alagoas, 57072-970 Maceió, AL, Brazil; c Chemistry Institute, Universidade de São Paulo, 05508-000 São Paulo-SP, Brazil; d Department of Chemistry, University of Malaya, 50603 Kuala Lumpur, Malaysia

## Abstract

In the title hydrate, C_16_H_15_BrO_2_SSe·H_2_O, the sulfinyl O atom lies on the opposite side of the mol­ecule to the Se and carbonyl O atoms. The benzene rings form a dihedral angle of 51.66 (17)° and are splayed with respect to each other. The observed conformation allows the water mol­ecules to bridge sulfinyl O atoms *via* O—H⋯O hydrogen bonds, generating a linear supra­molecular chain along the *b* axis; the chain is further stabilized by C—H⋯O contacts. The chains are held in place in the crystal structure by C⋯H⋯π and C—Br⋯π inter­actions.

## Related literature

For background to β,β-bis-substituted-carbonyl compounds, see: Reis *et al.* (2006 ▶). For related structures, see: Olivato *et al.* (2004 ▶); Zukerman-Schpector *et al.* (2009 ▶, 2010 ▶). For details of the synthetic protocols, see: Long (1946 ▶); Leonard & Johnson (1962 ▶); Zoretic & Soja (1976 ▶).
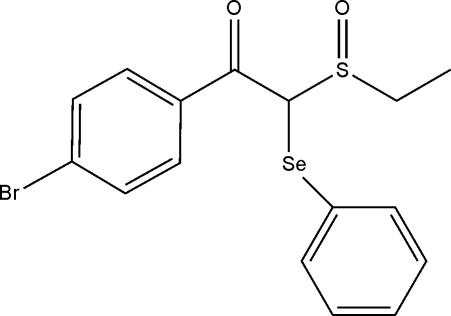


## Experimental

### 

#### Crystal data

C_16_H_15_BrO_2_SSe·H_2_O
*M* *_r_* = 448.23
Monoclinic, 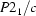

*a* = 14.6942 (2) Å
*b* = 6.1103 (1) Å
*c* = 21.5717 (4) Å
β = 113.714 (1)°
*V* = 1773.30 (5) Å^3^
*Z* = 4
Mo *K*α radiation
μ = 4.50 mm^−1^
*T* = 290 K
0.36 × 0.19 × 0.16 mm

#### Data collection

Nonius KappaCCD diffractometer
Absorption correction: multi-scan (*SADABS*; Sheldrick, 1996 ▶) *T*_min_ = 0.291, *T*_max_ = 0.734
32063 measured reflections
3734 independent reflections
3177 reflections with *I* > 2σ(*I*)
*R*_int_ = 0.076

#### Refinement

*R*[*F* ^2^ > 2σ(*F* ^2^)] = 0.037
*wR*(*F* ^2^) = 0.095
*S* = 1.03
3734 reflections
200 parameters
H-atom parameters constrained
Δρ_max_ = 0.80 e Å^−3^
Δρ_min_ = −0.55 e Å^−3^

### 

Data collection: *COLLECT* (Nonius, 1999 ▶); cell refinement: *SCALEPACK* (Otwinowski & Minor, 1997 ▶); data reduction: *DENZO* (Otwinowski & Minor, 1997 ▶) and *SCALEPACK*; program(s) used to solve structure: *SIR97* (Altomare *et al.*, 1999 ▶); program(s) used to refine structure: *SHELXL97* (Sheldrick, 2008 ▶); molecular graphics: *ORTEP-3* (Farrugia, 1997 ▶) and *DIAMOND* (Brandenburg, 2006 ▶); software used to prepare material for publication: *MarvinSketch* (Chemaxon, 2010 ▶) and *publCIF* (Westrip, 2010 ▶).

## Supplementary Material

Crystal structure: contains datablocks global, I. DOI: 10.1107/S1600536811012712/hg5022sup1.cif

Structure factors: contains datablocks I. DOI: 10.1107/S1600536811012712/hg5022Isup2.hkl

Additional supplementary materials:  crystallographic information; 3D view; checkCIF report

## Figures and Tables

**Table 1:** Hydrogen-bond geometry (Å, °) *Cg*1 and *Cg*2 are the centroids of the C5–C10 and C11–C16 rings, respectively.

*D*—H⋯*A*	*D*—H	H⋯*A*	*D*⋯*A*	*D*—H⋯*A*
O1w—H1w⋯O2^i^	0.85	1.95	2.788 (4)	169
O1w—H2w⋯O2	0.84	1.99	2.810 (4)	165
C2—H2⋯O1w^i^	0.98	2.40	3.334 (4)	159
C3—H3b⋯O1w^i^	0.97	2.54	3.434 (4)	153
C9—H9⋯O1w^ii^	0.93	2.55	3.320 (4)	141
C10—H10⋯O2^ii^	0.93	2.58	3.456 (4)	157
C14—H14⋯*Cg*1^iii^	0.93	2.96	3.793 (5)	149
C8—Br⋯*Cg*2^iv^	1.90 (1)	3.49 (1)	5.349 (3)	165 (1)

Symmetry codes: (i) 
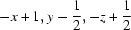
; (ii) 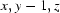; (iii) 
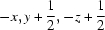
; (iv) 
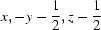
.

**Fig. 1.**
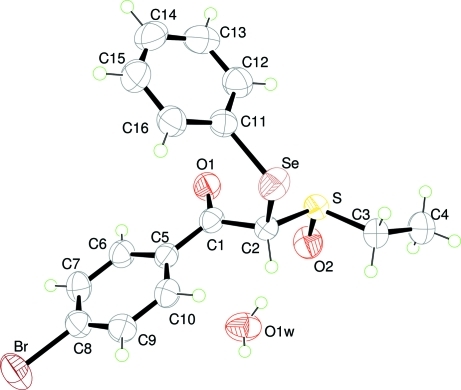
The molecular structure of (I) showing atom labelling scheme and displacement ellipsoids at the 35% probability level (arbitrary spheres for the H atoms).

**Fig. 2.**
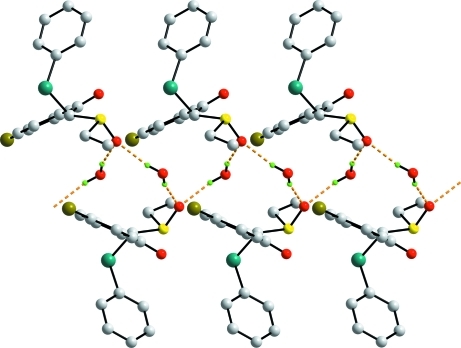
Supramolecular linear chain along the b axis in (I) mediated by O—H···O hydrogen bonding (orange dashed lines).

**Fig. 3.**
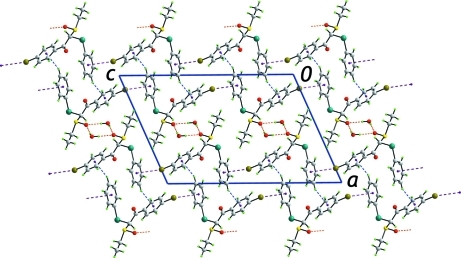
View of the unit-cell contents in projection down the b axis in (I). Chains shown in Fig. 2, sustained by O–H···O hydrogen bonding (orange dashed lines), are held in place by C—H···π and C—Br···π contacts, shown as blue and purple dashed lines, respectively.

## supplementary crystallographic information

### Comment

As part of our on-going research on the conformational and electronic interactions in some β,β-substituted-carbonyl compounds, *e.g.* 4'-substituted 2-(bromo)-2-(ethylsulfonyl)- and 4'-substituted 2-(methylthio)-2-(diethoxyphosphoryl)]-acetophenones, and 3,3-bis[(4'-chlorophenyl)thio]-1-methylpiperidin-2-one, using theoretical, spectroscopic and X-ray diffraction methods (Olivato *et al.*, 2004; Reis *et al.*, 2006; Zukerman-Schpector *et al.*, 2009; Zukerman-Schpector *et al.*, 2010), the title hydrate, (I), was synthesized and its crystal structure determined, Fig. 1.

With reference to the pyramidal-S atom, the sulfinyl-O lies to the opposite side of the molecule to each of the Se and carbonyl-O atoms. This conformation allows for the formation of supramolecular chains mediated by the sulfinyl-O and water molecules, see below. The benzene rings are splayed with respect to each other as seen in the value of the C1—C2—Se—C11 torsion angle of -27.7 (2) °; the dihedral angle formed between the rings is 51.66 (17) °.

In the crystal packing, the water molecules bridge sulfinyl-O atoms *via* O—H···O hydrogen bonds to form a linear supramolecular chain along the *b* axis, Fig. 2 and Table 1. Chains are stabilized by a series of C—H···O interactions, Table 1, and are held in place by C—H···π(aryl-Br) and C—Br···π(aryl-Se) interactions, Fig. 3 and Table 1.

### Experimental

Following the procedure of Long (1946), a solution of potassium hydroxide (400 mg, 7.2 mmol) and ethanothiol (0.5 ml, 7.2 mmol) in ethanol (10 ml) was added to a solution of 2-bromo-4'-bromoacetophenone (2.0 g, 7.2 mmol) in ethanol, to give 2-ethylthio-4'-bromoacetophenone (1.6 g, yield = 86%). The product was isolated and oxidized with 12 ml of an aqueous solution of sodium periodate (0.5 *M*) in acetonitrile (16 ml), after Leonard & Johnson (1962), to give 2-ethylsulfinyl-4'-bromoacetophenone that was extracted with dichloromethane and dried over anhydrous magnesium sulfate. 2-Ethylsulfinyl-4'-bromoacetophenone (730 mg, 2.6 mmol) was added drop-wise to a cooled (195 K) solution of diisopropylamine (0.4 ml, 2.6 mmol) and butyllithium (2.3 ml, 2.6 mmol) in THF (20 ml). After 20 minutes, phenylselenilbromide (610 mg, 2.6 mmol) dissolved in THF (10 ml) was added drop-wise to the enolate solution (Zoretic and Soja, 1976). After stirring for 3 h at 195 K, water (50 ml) was added at room temperature and extraction with chloroform was performed. The organic layer was dried over anhydrous magnesium sulfate. After evaporation of solvent, a crude solid was obtained. Purification through flash chromatography with a solution of hexane and ethyl acetate in a 1:1 ratio gave a mixture of the two possible diastereoisomers (500 mg, yield = 45%). One of the diastereoisomers was separated by recrystallization at low temperature (283 K) from chloroform. Suitable crystals for X-ray analysis were obtained by vapour diffusion of *n*-hexane into its chloroform solution at 283 K; *M*.pt. 366–367 K. IR (cm^-1^): ν(C=O) 1670, ν(S=O) 993. NMR (CDCl_3_, p.p.m.): δ 1.42–1.45 (3*H*, t ^3^J = 7.5 Hz), 2.92–2.99 (1*H*, dq, ^2^J = 13 Hz, ^3^J = 7.5 Hz), 3.32–3.25 (1*H*, dq, ^2^J = 13 Hz, ^3^J = 7.5 Hz), 5.44 (1*H*, s), 7.29–7.33 (2*H*, m, Aryl-H), 7.38–7.41 (1*H*, m, Aryl-H), 7.52–7.55 (2*H*, m, Aryl-H), 7.59–7.62 (2*H*, m, Aryl-H), 7.75–7.73 (2*H*, m, Aryl-H). Analysis found: C 42.76, H 3.84%. C_16_H_15_BrO_2_SSe.H_2_O requires: C 42.87, H 3.82%.

### Refinement

The H atoms were geometrically placed (C–H = 0.93–0.98 Å) and refined as riding with *U_iso_*(H) = 1.2–1.5*U_eq_*(C). Those of the water molecule were found in a difference map, fixed in those positions and refined with *U_iso_*(H) = 1.2*U_eq_*(O); see Table 1 for distances.

### Crystal data

**Table:**

C_16_H_15_BrO_2_SSe·H_2_O	*F*(000) = 888
*M**_r_* = 448.23	*D*_x_ = 1.679 Mg m^−^^3^
Monoclinic, *P*2_1_/*c*	Mo *K*α radiation, λ = 0.71073 Å
Hall symbol: -P 2ybc	Cell parameters from 23524 reflections
*a* = 14.6942 (2) Å	θ = 2.6–26.7°
*b* = 6.1103 (1) Å	µ = 4.50 mm^−^^1^
*c* = 21.5717 (4) Å	*T* = 290 K
β = 113.714 (1)°	Plate, colourless
*V* = 1773.30 (5) Å^3^	0.36 × 0.19 × 0.16 mm
*Z* = 4	

### Data collection

**Table:**

Nonius KappaCCD diffractometer	3734 independent reflections
Radiation source: sealed tube	3177 reflections with *I* > 2σ(*I*)
graphite	*R*_int_ = 0.076
CCD rotation images scans	θ_max_ = 26.7°, θ_min_ = 3.6°
Absorption correction: multi-scan (*SADABS*; Sheldrick, 1996)	*h* = −18→18
*T*_min_ = 0.291, *T*_max_ = 0.734	*k* = −7→7
32063 measured reflections	*l* = −27→25

### Refinement

**Table:**

Refinement on *F*^2^	Primary atom site location: structure-invariant direct methods
Least-squares matrix: full	Secondary atom site location: difference Fourier map
*R*[*F*^2^ > 2σ(*F*^2^)] = 0.037	Hydrogen site location: inferred from neighbouring sites
*wR*(*F*^2^) = 0.095	H-atom parameters constrained
*S* = 1.03	*w* = 1/[σ^2^(*F*_o_^2^) + (0.0428*P*)^2^ + 1.5141*P*] where *P* = (*F*_o_^2^ + 2*F*_c_^2^)/3
3734 reflections	(Δ/σ)_max_ < 0.001
200 parameters	Δρ_max_ = 0.80 e Å^−^^3^
0 restraints	Δρ_min_ = −0.55 e Å^−^^3^

### Special details

**Table:**

Geometry. All s.u.'s (except the s.u. in the dihedral angle between two l.s. planes) are estimated using the full covariance matrix. The cell s.u.'s are taken into account individually in the estimation of s.u.'s in distances, angles and torsion angles; correlations between s.u.'s in cell parameters are only used when they are defined by crystal symmetry. An approximate (isotropic) treatment of cell s.u.'s is used for estimating s.u.'s involving l.s. planes.
Refinement. Refinement of *F*^2^ against ALL reflections. The weighted *R*-factor *wR* and goodness of fit *S* are based on *F*^2^, conventional *R*-factors *R* are based on *F*, with *F* set to zero for negative *F*^2^. The threshold expression of *F*^2^ > 2σ(*F*^2^) is used only for calculating *R*-factors(gt) *etc*. and is not relevant to the choice of reflections for refinement. *R*-factors based on *F*^2^ are statistically about twice as large as those based on *F*, and *R*- factors based on ALL data will be even larger.

### Fractional atomic coordinates and isotropic or equivalent isotropic displacement parameters (Å^2^)

**Table:**

	*x*	*y*	*z*	*U*_iso_*/*U*_eq_	
C1	0.27316 (19)	0.2209 (5)	0.25810 (14)	0.0424 (6)	
C2	0.36493 (19)	0.1572 (5)	0.32017 (13)	0.0419 (6)	
H2	0.4114	0.0804	0.3055	0.050*	
C3	0.5393 (2)	0.2846 (6)	0.42271 (17)	0.0593 (8)	
H3A	0.5269	0.1896	0.4545	0.071*	
H3B	0.5683	0.1973	0.3977	0.071*	
C4	0.6102 (3)	0.4646 (8)	0.4605 (2)	0.0811 (12)	
H4A	0.5806	0.5522	0.4844	0.122*	
H4B	0.6243	0.5547	0.4290	0.122*	
H4C	0.6709	0.4014	0.4922	0.122*	
C5	0.23970 (19)	0.0756 (5)	0.19795 (14)	0.0409 (6)	
C6	0.1669 (2)	0.1548 (5)	0.13798 (15)	0.0488 (6)	
H6	0.1396	0.2925	0.1373	0.059*	
C7	0.1349 (2)	0.0315 (5)	0.07957 (16)	0.0544 (7)	
H7	0.0868	0.0854	0.0395	0.065*	
C8	0.1756 (2)	−0.1734 (5)	0.08164 (15)	0.0503 (7)	
C9	0.2473 (2)	−0.2565 (5)	0.14005 (16)	0.0506 (7)	
H9	0.2736	−0.3952	0.1405	0.061*	
C10	0.2796 (2)	−0.1304 (5)	0.19819 (15)	0.0470 (6)	
H10	0.3287	−0.1843	0.2379	0.056*	
C11	0.1967 (2)	0.0520 (5)	0.36178 (14)	0.0468 (6)	
C12	0.1754 (3)	0.2435 (6)	0.38661 (18)	0.0612 (8)	
H12	0.2261	0.3392	0.4116	0.073*	
C13	0.0772 (3)	0.2921 (7)	0.3739 (2)	0.0709 (10)	
H13	0.0618	0.4222	0.3900	0.085*	
C14	0.0026 (3)	0.1487 (8)	0.33776 (19)	0.0722 (10)	
H14	−0.0631	0.1809	0.3298	0.087*	
C15	0.0248 (3)	−0.0399 (8)	0.3137 (2)	0.0713 (10)	
H15	−0.0259	−0.1368	0.2894	0.086*	
C16	0.1217 (2)	−0.0899 (6)	0.32472 (17)	0.0577 (8)	
H16	0.1362	−0.2181	0.3073	0.069*	
O1	0.22999 (15)	0.3895 (3)	0.25913 (11)	0.0532 (5)	
O2	0.45208 (18)	0.5313 (4)	0.31552 (12)	0.0643 (6)	
O1W	0.4391 (2)	0.4022 (4)	0.18722 (14)	0.0729 (7)	
H1W	0.4651	0.2794	0.1849	0.088*	
H2W	0.4411	0.4158	0.2263	0.088*	
S	0.42388 (5)	0.40496 (12)	0.36491 (4)	0.04610 (18)	
Se	0.33094 (2)	−0.03515 (6)	0.380920 (17)	0.05674 (12)	
Br	0.13073 (3)	−0.34569 (7)	0.001765 (19)	0.07905 (15)	

### Atomic displacement parameters (Å^2^)

**Table:**

	*U*^11^	*U*^22^	*U*^33^	*U*^12^	*U*^13^	*U*^23^
C1	0.0385 (13)	0.0460 (15)	0.0428 (15)	−0.0001 (11)	0.0165 (11)	0.0043 (11)
C2	0.0366 (13)	0.0488 (15)	0.0393 (14)	0.0033 (11)	0.0143 (11)	−0.0005 (11)
C3	0.0458 (16)	0.077 (2)	0.0477 (17)	−0.0005 (15)	0.0116 (13)	−0.0088 (16)
C4	0.054 (2)	0.114 (3)	0.067 (2)	−0.015 (2)	0.0156 (18)	−0.030 (2)
C5	0.0351 (12)	0.0465 (14)	0.0402 (14)	−0.0005 (11)	0.0144 (11)	0.0018 (11)
C6	0.0438 (14)	0.0497 (16)	0.0474 (16)	0.0058 (12)	0.0127 (12)	0.0029 (12)
C7	0.0494 (16)	0.0604 (18)	0.0426 (16)	0.0017 (14)	0.0073 (13)	0.0028 (13)
C8	0.0491 (15)	0.0589 (18)	0.0440 (15)	−0.0071 (13)	0.0199 (13)	−0.0048 (13)
C9	0.0498 (15)	0.0490 (16)	0.0527 (17)	0.0008 (13)	0.0203 (13)	−0.0028 (13)
C10	0.0410 (14)	0.0505 (16)	0.0442 (15)	0.0017 (12)	0.0116 (12)	0.0036 (12)
C11	0.0484 (15)	0.0545 (16)	0.0403 (15)	−0.0053 (12)	0.0207 (12)	0.0044 (12)
C12	0.0638 (19)	0.062 (2)	0.0597 (19)	−0.0092 (16)	0.0271 (16)	−0.0088 (16)
C13	0.078 (2)	0.077 (2)	0.070 (2)	0.0102 (19)	0.043 (2)	−0.0010 (19)
C14	0.0531 (19)	0.109 (3)	0.061 (2)	0.003 (2)	0.0298 (17)	0.014 (2)
C15	0.0543 (19)	0.099 (3)	0.062 (2)	−0.0205 (19)	0.0248 (17)	−0.007 (2)
C16	0.0591 (18)	0.0643 (19)	0.0530 (18)	−0.0161 (15)	0.0260 (15)	−0.0090 (15)
O1	0.0507 (11)	0.0509 (11)	0.0526 (12)	0.0099 (9)	0.0149 (9)	−0.0012 (9)
O2	0.0661 (14)	0.0659 (14)	0.0611 (14)	−0.0174 (11)	0.0258 (12)	0.0026 (11)
O1W	0.0846 (17)	0.0675 (15)	0.0766 (17)	0.0192 (13)	0.0428 (14)	0.0114 (13)
S	0.0449 (4)	0.0504 (4)	0.0427 (4)	−0.0023 (3)	0.0172 (3)	−0.0049 (3)
Se	0.05056 (19)	0.0595 (2)	0.0579 (2)	0.00582 (13)	0.01947 (15)	0.01869 (14)
Br	0.0929 (3)	0.0816 (3)	0.0526 (2)	−0.0047 (2)	0.01875 (19)	−0.02037 (18)

### Geometric parameters (Å, °)

**Table:**

C1—O1	1.215 (3)	C8—Br	1.897 (3)
C1—C5	1.483 (4)	C9—C10	1.383 (4)
C1—C2	1.520 (4)	C9—H9	0.9300
C2—S	1.817 (3)	C10—H10	0.9300
C2—Se	1.969 (3)	C11—C12	1.375 (5)
C2—H2	0.9800	C11—C16	1.377 (4)
C3—C4	1.509 (5)	C11—Se	1.920 (3)
C3—S	1.809 (3)	C12—C13	1.388 (5)
C3—H3A	0.9700	C12—H12	0.9300
C3—H3B	0.9700	C13—C14	1.375 (6)
C4—H4A	0.9600	C13—H13	0.9300
C4—H4B	0.9600	C14—C15	1.357 (6)
C4—H4C	0.9600	C14—H14	0.9300
C5—C10	1.388 (4)	C15—C16	1.380 (5)
C5—C6	1.392 (4)	C15—H15	0.9300
C6—C7	1.378 (4)	C16—H16	0.9300
C6—H6	0.9300	O2—S	1.503 (2)
C7—C8	1.380 (4)	O1W—H1W	0.8525
C7—H7	0.9300	O1W—H2W	0.8362
C8—C9	1.374 (4)		
			
O1—C1—C5	122.1 (2)	C9—C8—Br	119.1 (2)
O1—C1—C2	119.1 (3)	C7—C8—Br	119.1 (2)
C5—C1—C2	118.8 (2)	C8—C9—C10	118.8 (3)
C1—C2—S	108.62 (19)	C8—C9—H9	120.6
C1—C2—Se	111.50 (17)	C10—C9—H9	120.6
S—C2—Se	109.77 (14)	C9—C10—C5	120.8 (3)
C1—C2—H2	109.0	C9—C10—H10	119.6
S—C2—H2	109.0	C5—C10—H10	119.6
Se—C2—H2	109.0	C12—C11—C16	120.4 (3)
C4—C3—S	109.2 (3)	C12—C11—Se	121.9 (2)
C4—C3—H3A	109.8	C16—C11—Se	117.6 (2)
S—C3—H3A	109.8	C11—C12—C13	119.2 (3)
C4—C3—H3B	109.8	C11—C12—H12	120.4
S—C3—H3B	109.8	C13—C12—H12	120.4
H3A—C3—H3B	108.3	C14—C13—C12	120.2 (4)
C3—C4—H4A	109.5	C14—C13—H13	119.9
C3—C4—H4B	109.5	C12—C13—H13	119.9
H4A—C4—H4B	109.5	C15—C14—C13	119.9 (3)
C3—C4—H4C	109.5	C15—C14—H14	120.0
H4A—C4—H4C	109.5	C13—C14—H14	120.0
H4B—C4—H4C	109.5	C14—C15—C16	120.8 (3)
C10—C5—C6	118.9 (3)	C14—C15—H15	119.6
C10—C5—C1	123.3 (2)	C16—C15—H15	119.6
C6—C5—C1	117.7 (2)	C11—C16—C15	119.4 (3)
C7—C6—C5	120.8 (3)	C11—C16—H16	120.3
C7—C6—H6	119.6	C15—C16—H16	120.3
C5—C6—H6	119.6	H1W—O1W—H2W	108.1
C6—C7—C8	118.8 (3)	O2—S—C3	104.37 (15)
C6—C7—H7	120.6	O2—S—C2	105.07 (13)
C8—C7—H7	120.6	C3—S—C2	98.16 (14)
C9—C8—C7	121.8 (3)	C11—Se—C2	101.82 (11)
			
O1—C1—C2—S	−28.1 (3)	C16—C11—C12—C13	0.1 (5)
C5—C1—C2—S	151.4 (2)	Se—C11—C12—C13	−176.7 (3)
O1—C1—C2—Se	93.0 (3)	C11—C12—C13—C14	0.9 (5)
C5—C1—C2—Se	−87.5 (2)	C12—C13—C14—C15	−0.8 (6)
O1—C1—C5—C10	−171.6 (3)	C13—C14—C15—C16	−0.3 (6)
C2—C1—C5—C10	8.9 (4)	C12—C11—C16—C15	−1.1 (5)
O1—C1—C5—C6	10.6 (4)	Se—C11—C16—C15	175.8 (3)
C2—C1—C5—C6	−168.9 (2)	C14—C15—C16—C11	1.3 (6)
C10—C5—C6—C7	−0.2 (4)	C4—C3—S—O2	64.1 (3)
C1—C5—C6—C7	177.7 (3)	C4—C3—S—C2	172.0 (3)
C5—C6—C7—C8	0.7 (5)	C1—C2—S—O2	−61.3 (2)
C6—C7—C8—C9	−0.5 (5)	Se—C2—S—O2	176.53 (14)
C6—C7—C8—Br	179.2 (2)	C1—C2—S—C3	−168.7 (2)
C7—C8—C9—C10	−0.2 (5)	Se—C2—S—C3	69.18 (16)
Br—C8—C9—C10	−179.9 (2)	C12—C11—Se—C2	−76.2 (3)
C8—C9—C10—C5	0.7 (4)	C16—C11—Se—C2	106.9 (2)
C6—C5—C10—C9	−0.5 (4)	C1—C2—Se—C11	−27.7 (2)
C1—C5—C10—C9	−178.3 (3)	S—C2—Se—C11	92.67 (15)

### Hydrogen-bond geometry (Å, °)

**Table:**

Cg1 and Cg2 are the centroids of the C5–C10 and C11–C16 rings, respectively.

**Table:**

*D*—H···*A*	*D*—H	H···*A*	*D*···*A*	*D*—H···*A*
O1w—H1w···O2^i^	0.85	1.95	2.788 (4)	169
O1w—H2w···O2	0.84	1.99	2.810 (4)	165
C2—H2···O1w^i^	0.98	2.40	3.334 (4)	159
C3—H3b···O1w^i^	0.97	2.54	3.434 (4)	153
C9—H9···O1w^ii^	0.93	2.55	3.320 (4)	141
C10—H10···O2^ii^	0.93	2.58	3.456 (4)	157
C14—H14···Cg1^iii^	0.93	2.96	3.793 (5)	149
C8—Br···Cg2^iv^	1.897 (3)	3.4921 (16)	5.349 (3)	165.34 (10)

Symmetry codes: (i) −*x*+1, *y*−1/2, −*z*+1/2; (ii) *x*, *y*−1, *z*; (iii) −*x*, *y*+1/2, −*z*+1/2; (iv) *x*, −*y*−1/2, *z*−1/2.
